# A potential biomarker for age-related macular degeneration disease: iris freckles

**DOI:** 10.1186/s40942-024-00575-z

**Published:** 2024-09-05

**Authors:** Hakan Koc, Seda Uzunoğlu

**Affiliations:** Faculty of Medicine, Department of Ophthalmology, Giresun University, Giresun, 28100 Turkey

**Keywords:** Iris freckles, Age-related macular degeneration, Ultraviole exposure

## Abstract

**Backgraund:**

To determine the potential relationship between age-related macular degeneration and iris freckles.

**Method:**

In this case-control study, iris photographs of 300 eyes of 300 patients diagnosed with age-related macular degeneration and 300 eyes of 300 healthy volunteers were obtained with the help of a high-resolution mobile phone camera. The evaluated iris photographs were classified according to the Descriptive Iris Color Classification Scale.

**Results:**

The average age of the AMD group is 73.05 ± 6.93, and the average age of the control group is 73.43 ± 5.72. (*p* = 0.124) While freckles were present in 200 (66.7%) of the patients in the AMD group, freckles were not observed in 100 patients (33.3%) of AMD group. While freckles were present in 142 (47.3%) of the patients in the control group, freckles were not observed in 158 of control group(52.7%). There was a significant difference in the presence of freckles between the two groups. (*p* < 0.001) The average number of freckles in the AMD group was 3.97 ± 3.07, and the number of freckles in the control group was 3.06 ± 2.55. (*p* = 0.001)

**Conclusion:**

We think that evaluation of iris details, especially the presence of iris freckles, should be used routinely in age-related macular degeneration screening. The risk of age-related macular degeneration can be predicted by evaluating iris details, which is an easy and inexpensive method.

## Introduction

Age-related macular degeneration (AMD) is a progressive, chronic disease of the central retina (macula) and the leading cause of vision loss worldwide [1]. Age is the main risk factor for the development of AMD. However, smoking, exposure to sunlight, increased body mass index, alcohol consumption, hypertension, previous cataract surgery, lipid profile disorder, genetic factors, and oxidative stress are the main risk factors [2]. It is seen as a risk factor in the development of AMD for patients who are exposed to sunlight for long periods of time throughout their lives. Visible light and ultraviolet (UV) are considered risk factors for AMD [3]. In a meta-analysis study evaluating the relationship between sunlight and age-related macular diseases, it was shown that the risk of AMD increased significantly in individuals exposed to higher levels of sunlight [4]. Similarly, Qu et al. reported that exposure to ultraviolet radiation increased the risk of AMD in a study of 19,707 patients [5].

Iris freckles are the most common melanocytic iris lesions. Iris freckles are discrete, superficial, atypical colonies of melanocytes on the iris surface that differ in their ability to synthesize pigment. The most important feature that distinguishes iris freckles from iris nevus is that iris freckles do not involve the iris stroma and are on the surface of the iris. Although they are common in adults, little is currently known about the occurrence of iris freckles. Iris freckles are more common in older people than in young adults. The UV spectrum of sunlight is a well-known promoter of melanogenesis. Eyes are exposed to light most of the time, so it is obvious that the iris is also affected by sunlight [6–8]. Additionally, Grigore et al. reported a higher rate of iris freckles in patients with ultraviolet-associated skin cancer [9].

Since the eyes are exposed to light for long periods of time, the iris is also affected by sunlight. Furthermore, high cumulative doses of sunlight are a known risk factor for macular degeneration.For these reasons, there is suspicion that there may be a relationship between iris freckles and age-related macular degeneration. Schwab et al. emphasized that iris freckles are associated with chronic sunlight exposure and that evaluation of iris freckles may also help in understanding the role of sunlight in various ophthalmological diseases [10].

The aim of this study was to evaluate the relationship between iris freckles and age-related macular degeneration, which are thought to be associated with sunlight exposure. Also, to investigate whether iris freckles could be a potential biomarker for age-related macular degeneration.

## Materials and methods

### Ethics committee approval

Ethics committee approval was received for this study from the Giresun Training and Research Hospital Local Ethics Committee (IRB No 24.04.2024/05). Informed consent was obtained from all participants before inclusion in the study. The study was conducted in accordance with the Declaration of Helsinki.

### Study type and selection of patients

A case-control study was conducted at the Giresun Training and Research Hospital Ophthalmology Clinic with 600 patients, divided into two groups: 300 patients with age-related macular degeneration and 300 healthy volunteers without age-related macular degeneration. All eyes underwent a full ophthalmologic examination, including refractive measurements with an autorefractor (Topcon Auto Ref-Keratometer, Tokyo, Japan), best-corrected visual acuity (BCVA) assessment, intraocular pressure measurements, slit-lamp examination, biomicroscopic evaluation, and SD-OCT (Retinascan Advanced RS-3000; NIDEK, Gamagori, Japan). Patients with age-related macular degeneration were divided into subgroups according to Fundus Fluorescein Angiography and Spectral Domain Optical Coherence Tomography (SD-OCT) results. (Early AMD, Middle AMD, Late AMD) Early AMD included patients with medium sized drusen (> 63 μm and ≤ 125 μm), middle AMD included patients with large drusen (> 125 μm) and intraretinal fluid, subretinal fluid and serous pigment epithelial detachment without neovascularization and late AMD included patients with geographic atrophy and neovascularization [11, 12]. If age-related macular degeneration was not detected in the detailed ophthalmologic examination, they were included in the healthy control group. Considering that outcome measures from both eyes of the same subject tend to be positively correlated, the right eye was selected for study measures if both eyes were eligible for inclusion.

Patients with corneal diseases that would prevent clear understanding of iris details, medications that would affect iris color structure, or surgical procedures that could cause trauma to the iris were excluded from the study.

### Exclusion criteria

- History of ocular surgery.
- History of ocular trauma.
- Keratitis or corneal degeneration.
- Those receiving antiglaucomatous drug treatment (especially the use of prostaglandin analogues).
- Use of drugs with known corneal toxicity.
- Systemic drug use that may affect the iris structure.
- Advance Dry Eye.

### Obtaining iris photographs

Digital images of each patient’s right eye, without pupil dilation, were taken under the same conditions with the same equipment by two authors trained by a professional photographer. Iris photographs were taken using the high-resolution camera system of the I Phone 15 Pro (Apple Inc., California, U.S.A.). All photos were taken using standard settings to obtain high-quality digital images without being affected by external lighting conditions. After high-resolution images with iris details were obtained, the obtained images were transferred to a digital support with the AirDrop feature using the AirDrop feature of the mobile phone and stored in separate folders for each patient.

### Evaluation of iris photographs

The resulting digital images of the iris were evaluated on a computer (MacBook Air 2020 M1 chip) monitor set to a native resolution of 2560 × 1600 at a density of 227 pixels per inch using the MacOS photo viewer program. The evaluated iris images were classified according to the Descriptive Iris Color Classification Scale [13]. The classification was made by two experienced ophthalmologists.

The ophthalmologists who evaluated the iris photographs are assistant professor and specialist. Iris photographs were taken in the same room, in the same light, with the same cell phone, at the same resolution, at the same distance, using flash light in all patients. The evaluation of iris photographs was performed on the same computer, using the same screen light brightness and magnification.

This scale consists of three parameters:

1. **Periphery**: blue-gray/green/hazel/light brown/dark brown.
2. **Colarette**: blue/light brown/dark brown.
3. **Iris freckles**: absent / present.

**Fig. 1:**
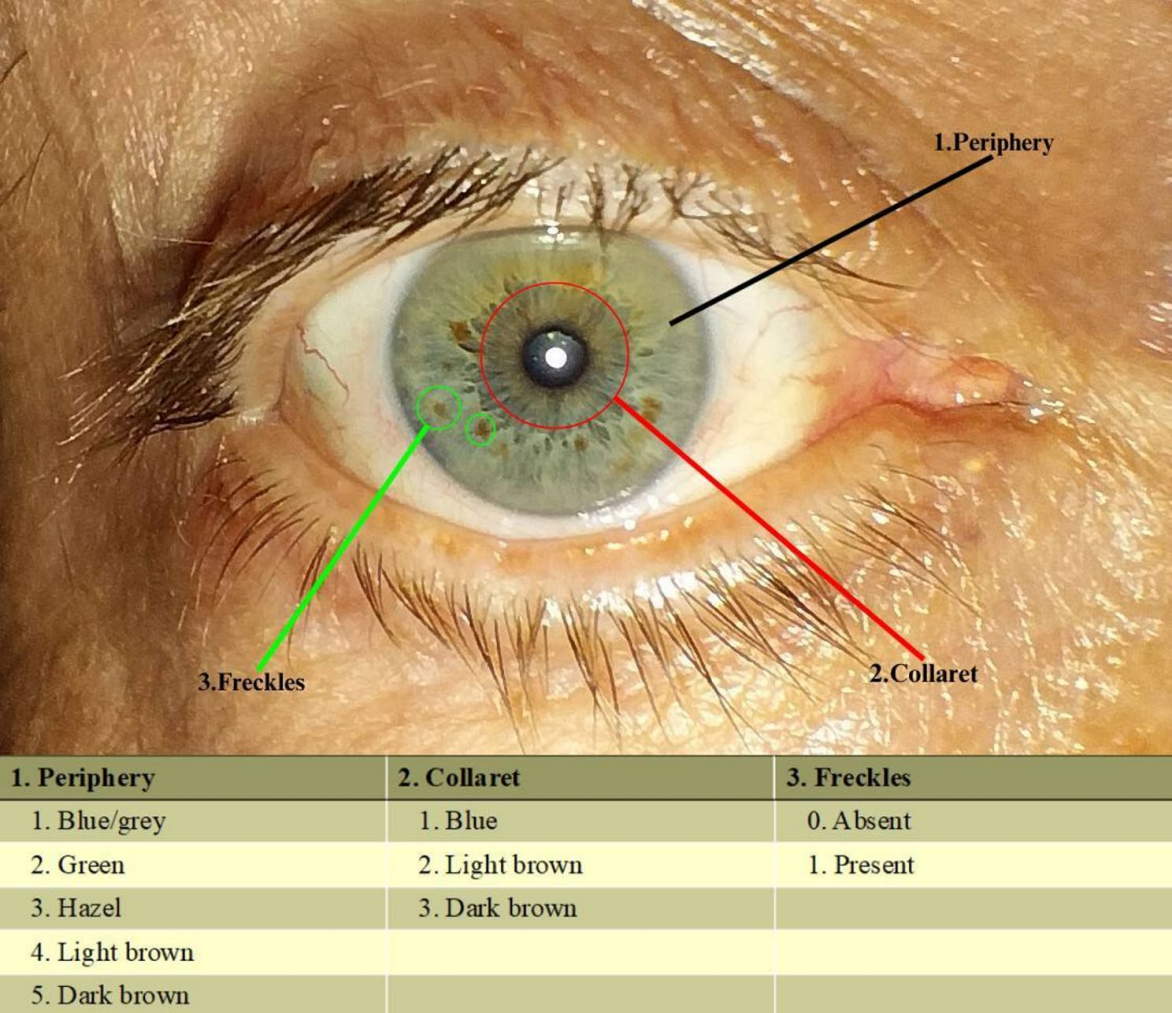
The descriptive iris colour classification scale

An example of the Iris Color Classification Scale of the patients participating in the study is shown in Fig. 1.

Although not included in this scale, the number of iris freckles and the location of the iris freckles (upper nasal, upper temporal, lower nasal, and lower temporal) were also recorded.

### Statistical analysis

Statistical analyses were performed using SPSS 26.0 for Windows (IBM Corp., Armonk, NY, USA). The suitability of the variables for a normal distribution was evaluated visually (histograms and probability graphs) and analytically (Kolmogorov-Smirnov/Shapiro-Wilk test). Quantitative variables between the two groups were compared using Student’s t/Mann-Whitney tests. Qualitative variables between groups were compared using Chi-square/Fisher exact tests. The Spearman rank correlation coefficient was calculated to evaluate the relationship between the number of iris freckles and age. A probability level of *P* < 0.05 was considered statistically significant.

## Results

There were 300 patients in the AMD group and 300 healthy volunteers in the control group. The average age of the AMD group is 73.05 ± 6.93, and the average age of the control group is 73.43 ± 5.72. (*p* = 0.124) In the AMD group, 144 of the patients were female (48%) and 156 were male (52%). Of the healthy volunteers in the control group, 147 were women (47%), and 153 were men (51%). (*p* = 0.806)

**Fig. 2:**
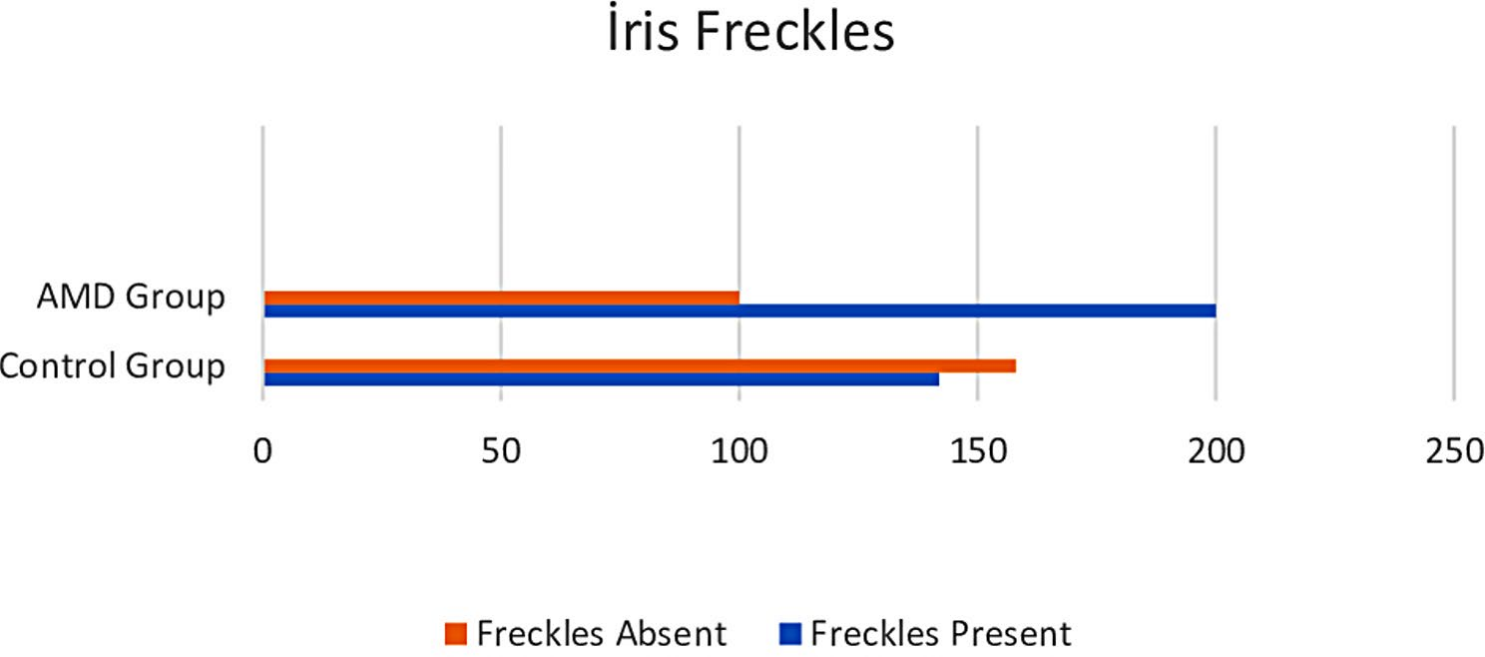
Distribution of iris freckles according to location in AMD and Control groups

In the AMD group, 200 patients (66.7%) had freckles and 100 patients (33.3%) did not have freckles. In the control group, 142 patients (47.3%) had freckles and 158 patients (52.7%) did not have freckles (Fig. 2). There was a significant difference in the presence of freckles between the two groups. (*p* < 0.001) While freckles were present in 167 (57.4%) of the male patients, freckles were not observed in 124 patients (42.6%). While freckles were present in 175 (56.6%) of the female patients, freckles were not observed in 134 patients (43.4%). No significant difference was observed between genders in terms of freckles. (*p* = 0.852) When evaluated according to the location of the freckles in AMD patients, they were observed in 10 upper temporal, 96 lower temporal, 33 lower nasal, and 61 multiple regions. According to the location of the freckles, it was most frequently seen in the lower temporal region and was statistically significant. (*p* < 0.001)

**Fig. 3:**
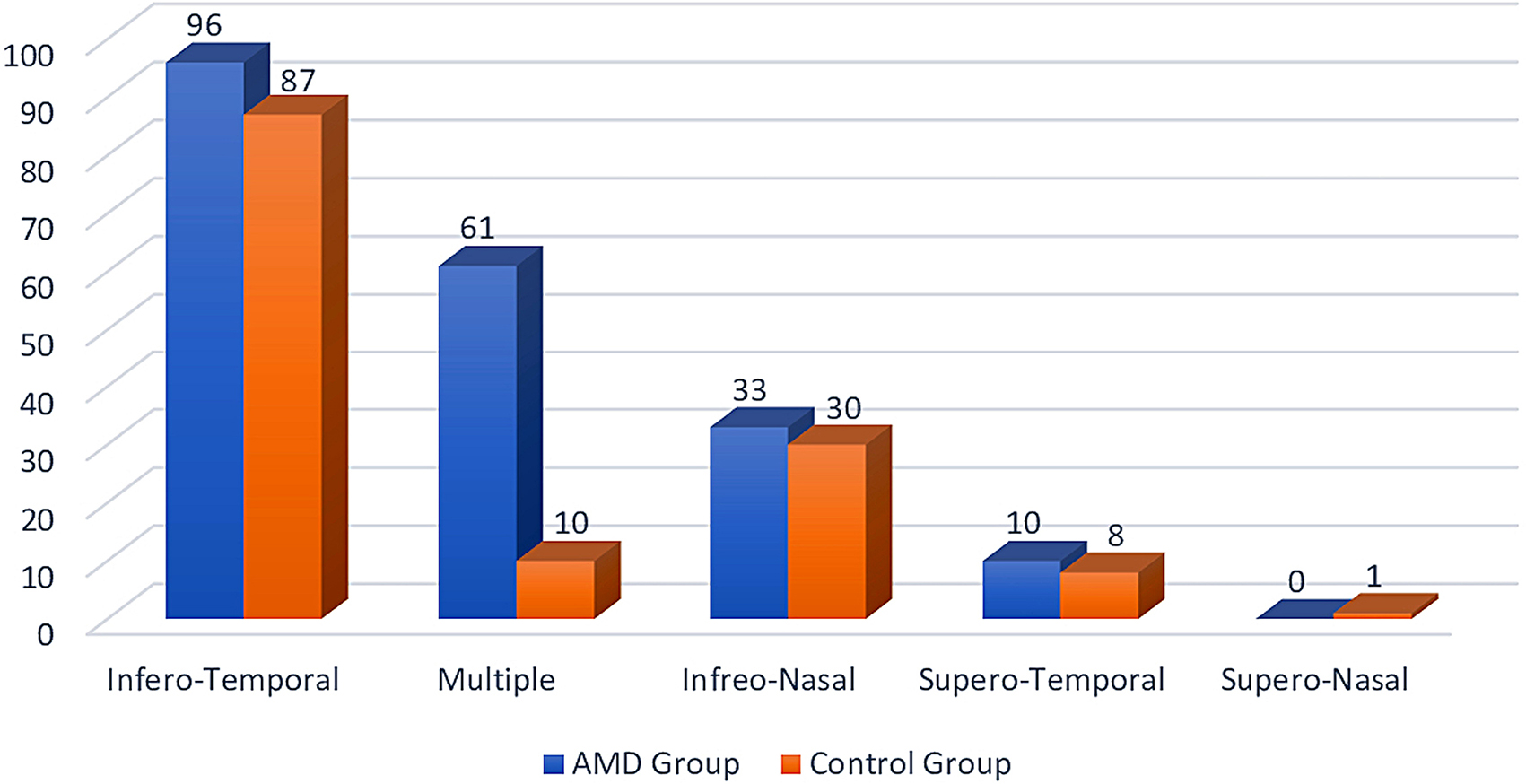
Distribution of iris freckles in AMD and Control groups

When evaluated according to the location of the freckles in the control group, it was observed in 1 upper nasal, 8 upper temporal, 87 lower temporal, 30 lower nasal, and 10 multiple regions. Freckles were most frequently seen in the lower temporal region, according to location, and were statistically significant. (*p* < 0.001) The distribution of iris freckles according to their location in the AMD and control groups is shown in Fig. 3.

The average number of freckles was 3.97 ± 3.07 in the AMD group, and the average number of freckles was 3.06 ± 2.55 in the control group. The difference in the average number of freckles between the two groups was statistically significant (*p* = 0.001).

No significant correlation was observed between age and the number of freckles (*r* = 0.026 *p* = 0.48).

While freckles were observed in 224 of the individuals with light eye color (periphery 1, 2, 3, 4), freckles were not observed in 43 of them. While freckles were observed in 118 of the individuals with dark eye color (periphery 5), freckles were not observed in 215. The relationship between eye color and the presence of freckles was statistically significant. (*p* < 0.001). The presence of freckles was higher in individuals with light-colored eyes than in individuals with dark-colored eyes. While the number of iris periphery 2 patients in AMD patients was 37, it was 22 in the control group, and the difference was found to be statistically significant. (*p* = 0.04) While 50 people in the control group had an iris periphery number of 5, it was found in 19 of the AMD patients, and the difference was found to be statistically significant. (*p* < 0001). While the number of iris collaret 2 patients in AMD patients was 207, it was 146 in the control group, and the difference was found to be statistically significant. (*p* < 0.001) Iris collaret 3 was present in 123 patients in the control group and in 55 patients in the AMD group, and the difference was found to be statistically significant. (*p* < 0.001). All of these data are shown in Tables 1 and 2.

**Table 1:** Distribution of iris freckles

Parameter	Iris Freckles Present Absent		*P* value
	*n* %	*n* %	
**AMD Group** **-Early AMD** *Medium drusen (> 63 μm and ≤ 125 μm) and no AMD pigmentary abnormalities **-Intermediate AMD** *Large drusen (> 125 μm) or medium drusen (> 63 μm) in addition to AMD pigmentary abnormalities *Intraretinal fluid in the absence of neovascularisation *Subretinal fluid in the absence of neovascularisation *Serous pigment epithelial detachment (PED) without neovascularisation **-Late AMD** *Geographic atrophy *Neovascular AMD **Control Group**	**200 66**,**7** **68 22**,**66** 68 22,66 **86 28**,**66** 28 9,33 26 8,66 16 5,33 16 5,33 **46 15**,**33** 25 8,33 21 7 **142 47**,**3**	**100 33**,**3** **31 10**,**33** 31 10,33 **35 11**,**66** 10 3,33 10 3,33 8 2,66 7 2,33 **24 8** 13 4,33 11 3,66 **158 52**,**7**	< 0,001*
**Sex** Female Male	134 43,4 124 42,6	175 56,6 167 57,4	0,852**

* Statistical difference of iris freckles between AMD group and Control group

** Statistical difference between genders

**Table 2:** Demographic charecteristics and distribution of iris pattern

Charecteristic	AMD Group *n* = 300	Control Group *n* = 300	*p*-Value
Sex, (%) Female Male Total	156 (52,0) 144 (48,0) 300	153 (51,0) 147 (49,0) 300	0,806
Age (years), mean (SD)	73,05 (± 6,93)	73,43 (± 5,72)	0,124
Freckles number (SD)	3,97 (3,07)	3,06 (2,55)	0,001
Iris periphery color, n (%) Blue/grey Green Hazel Light Brown Dark Brown	39 (13,1) 37 (12,3) 76 (25,3) 129 (43,0) 19 (6,3)	31 (10,3) 22 (7,3) 62 (20,7) 135 (45,0) 50 (16,7)	0,309 0,040 0,174 0,622 < 0,001
Iris collaret color, n (%) Blue Light Brown Dark Brown	38 (12,7) 207 (69,0) 55 (18,3)	31 (10,3) 146 (48,7) 123 (41,0)	0,370 < 0,001 < 0,001
Freckles location, n (%) Upper Nasal Upper Temporal Lower Temporal Lower Nasal Multipl Location	0 (0,0) 10 (5,0) 96 (48,0) 33 (16,5) 61 (30,5)	1 (0,7) 8 (5,6) 87 (61,3) 30 (21,1) 16 (11,3)	0,415 0,810 0,016 0,322 < 0,001

N: the total number of individuals or observations in the sample, SD: standard deviation,

## Discussion

This study is the first to evaluate the relationship between iris freckles and ocular pathologies. We evaluated the relationship between iris freckles and age-related macular degeneration, which is thought to be associated with ultraviolet exposure. This study showed that the presence of iris freckles was statistically significant in the age-related macular degeneration group compared with the control group. According to these results, we think that the evaluation of iris pattern, especially iris freckles, may be a potential biomarker in determining age-related macular degeneration disease.

Exposure to low doses of ultraviolet light stimulates pigmentation, a protective response mediated by melanocytes located in the epidermis. UV exposure causes DNA damage in the epidermis. As a result, melanocytes cause the synthesis of melanin, which easily absorbs ultraviolet light, and its transfer to neighboring keratinocytes. All of these conditions result in increased skin pigmentation after exposure [14]. The formation of iris freckles caused by ultraviolet exposure on the anterior superficial layer of the iris is similar to this mechanism. Iris freckles are an acceleration of melanocytes containing large melanin granules on the front surface of the iris, one of the most sun-exposed areas of the body [6, 7]. Schwab et al. reported that the formation of iris freckles is induced by sunlight, and although iris freckles are not believed to have malignant potential, their presence may be indicative of a high cumulative dose [10].

In our study, it was determined that the presence of iris freckles and the average number of iris freckles were significantly higher in age-related macular degeneration patients compared to the control group. The association between AMD and iris freckles may be due to apoptosis induced by ultraviolet exposure. The UV-B spectrum of sunlight directly causes DNA damage [15]. After high doses of UV-B, the DNA repair mechanism triggers appoptosis to eliminate unrepaired cells. It also stimulates melanocytic stimulation to prevent future damage. Melanocytes produce melanin, a photoprotectant that readily absorbs UV light [16]. Similarly, iris freckles are clusters of melanocytes containing large melanin granules on the anterior surface of the iris most exposed to the ultraviolet spectrum of sunlight [6]. Although the pathogenesis of AMD has not been clearly established, it has been shown that UV exposure of ocular tissues can cause cellular modifications and retinal pigment epithelial apoptosis following photochemical reactions. We think that the relationship between UV exposure, iris freckles and AMD may be due to these reasons.

This suggests that an assessment of the presence of iris freckles associated with ultraviolet exposure can be used as a biomarker for age-related macular degeneration. We think that it can be used as a simple method to evaluate the iris and determine the risk of age-related macular degeneration by using only a light source, without performing a fundus examination. When the results of this study and the results of our study are evaluated together, it is seen that the iris pattern in epidermal skin cancer and the iris pattern in age-related macular degeneration have similar characteristics. These results provide strong evidence that a light-colored iris is more sensitive to ultraviolet light and that iris freckles are associated with ultraviolet exposure.

Iris freckles were seen to be more common in patients with light eye color in both groups. This may indicate that light iris colors are more photosensitivity than dark iris colors. Our results are parallel to the study by Sample et al., in which they reported that nevus, freckles, and melanomas may occur due to both individual risk factors (light iris/skin color, etc.) and environmental risk factors (ultraviolet exposure, etc.) [17]. It should also be kept in mind that it may be more difficult to distinguish iris freckles in dark-colored irises than in light-colored irises. Grigore et al., in their study evaluating iris patterns in patients with epidermal skin cancers caused by ultraviolet exposure, reported that the rate was highest in the presence of iris freckles with light-colored eyes and lowest in the absence of iris freckles with dark-colored iris [9]. When the results of this study and the results of our study were evaluated together, it was seen that the iris pattern in epidermal skin cancer and the iris pattern in age-related macular degeneration had similar characteristics. These results provide strong evidence that a light-colored iris is more sensitive to ultraviolet light and that iris freckles are associated with ultraviolet exposure. In our study, especially the presence of green periphery and light brown colorette and iris freckles showed the highest statistical association with age-related macular degeneration compared to the control group. These results indicate that the presence of green periphery, a light brown colorette and iris freckles is the highest risk for age-related macular degeneration. In addition, the presence of dark brown colorectum with dark brown iris and the absence of iris freckles were found to be statistically significantly lower in age-related macular degeneration. This indicates that the absence of iris freckles combined with a dark iris pattern has the lowest risk for age-related macular degeneration.

Iris freckles locations in both the control group and the AMD group, respectively: lower temporal, lower nasal, upper temporal, and upper nasal quadrant. Since the inferior temporal quadrant of the iris is the most vulnerable area to ultraviolet light, we think that the density of iris freckles is highest in this quadrant. We predict that the upper nasal quadrant is least exposed to ultraviolet light because it is surrounded by the superior and medial walls of the orbit, and therefore the location of the iris freckles is least in the upper nasal quadrant. Considering that the upper temporal quadrant is protected only by the orbital superior and the lower nasal quadrant is protected only by the orbital medial wall, the presence of more iris freckles in these quadrants than the upper nasal quadrant confirms our hypothesis.

In our study, the increase in the presence and number of freckles was not correlated with age. Our study shows that the presence of freckles does not actually occur with age, and the number of iris freckles does not increase with age but is related to the duration of ultraviolet exposure. This suggests that the duration of ultraviolet exposure for patients is more important than age in the formation of iris freckles.

The limitations of our study are that the number of patients is relatively small and the rate of iris freckles in the control group is slightly less than the number described in the literature, due to the low number of sunny days in the region where we studied (Giresun, Black Sea Region, Turkey).[5, 6] (60%-46%) However, the fact that the rate of iris freckles in the AMD group is higher than the rate reported in the literature adds additional importance to our study. (60–66.7%) Due to the relationship between the palpebral fissure distances of the patients and ultraviolet exposure, not evaluating the palpebral fissure distances of the patients participating in the study is one of the limitations of the study. In addition, the use of sunglasses by the patients was not included in our study.

## Conclusion

In conclusion, the presence of iris freckles can be used as a potential biomarker for age-related macular degeneration. In particular, we consider the presence of green periphery, light brown colorette, and iris freckles as a potential risk factor for age-related macular degeneration. Iris color classification, which is an easy and inexpensive method, can provide information about a potential risk for age-related macular degeneration at first glance. Therefore, we strongly support routine use by ophthalmologists of assessment of iris details, particularly the presence of iris freckles, in screening for age-related macular degeneration.

## Data Availability

No datasets were generated or analysed during the current study.
